# Examining Health Care Provider Experiences With Patient Portal Implementation: Mixed Methods Study

**DOI:** 10.2196/65967

**Published:** 2025-01-31

**Authors:** Shipra Taneja, Kamini Kalia, Terence Tang, Walter P Wodchis, Shelley Vanderhout

**Affiliations:** 1 Trillium Health Partners Institute for Better Health Mississauga, ON Canada; 2 Trillium Health Partners Mississauga, ON Canada; 3 Management and Evaluation Institute of Health Policy University of Toronto Toronto, ON Canada

**Keywords:** patient portal, mixed methods, implementation, healthcare provider, health system, patient care, online questionnaire, Canada, descriptive statistics, thematic analysis

## Abstract

**Background:**

Health systems are increasingly offering patient portals as tools for patients to access their health information with the goal of improving engagement in care. However, understanding health care providers’ perspectives on patient portal implementation is crucial.

**Objective:**

This study aimed to understand health care providers’ experiences of implementing the MyChart patient portal, perspectives about its impact on patient care, clinical practice, and workload, and opportunities for improvement.

**Methods:**

Using an explanatory sequential mixed methods approach, we conducted a web-based questionnaire and semistructured individual interviews with health care providers at a large Canadian community hospital, 6 months after MyChart was first offered to patients. We explored perspectives about the impact of MyChart on clinical practice, workload, and patient care. Data were analyzed using descriptive statistics and thematic analysis.

**Results:**

In total, 261 health care providers completed the web-based questionnaire, and 15 also participated in interviews. Participants agreed that patients should have access to their health information through MyChart and identified its benefits such as patients gaining a greater understanding of their own health, which could improve patient safety (160/255, 62%). While many health care providers agreed that MyChart supported better patient care (108/258, 42%), there was limited understanding of features available to patients and expectations for integrating MyChart into clinical routines. Concerns were raised about the potential negative impacts of MyChart on patient-provider relationships because sensitive notes or results could be inappropriately interpreted (109/251, 43%), and a potential increase in workload if additional portal features were introduced. Suggested opportunities for improvement included support for both patients and health care providers to learn about MyChart and establishing guidelines for health care providers on how to communicate information available in MyChart to patients.

**Conclusions:**

While health care providers acknowledged that MyChart improved patients’ access to health information, its implementation introduced some friction and concerns. To reduce the risk of these challenges, health systems can benefit from engaging health care providers early to identify effective patient portal implementation strategies.

## Introduction

Providing patients with access to their health data through patient portals has become an important focus for health care systems [1]. These portals are designed to strengthen patient and provider relationships, foster greater patient engagement, improve patient education, and ultimately improve patient care [2-4]. Offering various functionalities such as appointment reminders and scheduling, access to notes and test results, and prescription refills, patient portals can equip patients to manage their health and care [5]. This increased autonomy is intended to allow them to actively participate in shared decision-making with their health care team [6,7].

While patients and families desire access to patient portals and have reported benefits of using them [8], health systems have faced challenges in implementing them. These challenges include integrating portals into existing technological infrastructure and clinical workflows [6,9]. In addition, health care providers have expressed concerns about the increased workload associated with managing patient portal inquiries and the potential for information overload and misinterpretation by patients [3,8,10,11]. This is particularly concerning in the post–COVID-19 era, where health care providers are already facing burnout due to increased patient complexity and staffing shortages [12]. Moreover, there is often a lack of agreement between patients and health care providers about which features, and content should be accessible through patient portals, leading to potential misalignment and confusion [3].

Many hospitals have adopted or are in the process of adopting digital health information systems with accompanying patient portals. While patient portals are primarily designed for patients and their families, the acceptance and influence of health care providers can significantly impact the adoption of these digital health tools [9,13]. Their endorsement can shape patient and caregiver attitudes, influencing whether they perceive the portal as a worthwhile investment of time and effort to learn and use [14-16]. As health care providers are at the forefront of care and are encouraged to increase patient engagement, it is crucial to understand their perspectives to best improve how patient portals can support patient and health care provider needs. Our objective was to understand and describe the initial impact of a patient portal on patient care and health care provider workload and suggest areas for improvement.

## Methods

### Setting and Implementation

This study was conducted at Trillium Health Partners (THP), a large community hospital comprised of 3 sites in Mississauga, Ontario, Canada. THP employs over 11,414 physicians, nurses, and allied health professionals, and provides care to a diverse population in the Mississauga and West Toronto area. During 2023-2024, THP had over 1.7 million patient visits and had 1457 inpatient beds [17]. This evaluation represents a strategic partnership to explore the initial impacts of implementing a vendor-developed patient portal (MyChart) across THP.

MyChart is the patient portal provided by Epic, a digital electronic health record used in health systems across the world [4], which was implemented at THP on October 10, 2020. MyChart was made available in English at THP in September 2023 to all patients aged 12 years or older. For children aged 12 years or younger, parent or guardian proxy access was provided as requested. At the time of this study, 35,387 patients had activated MyChart accounts. MyChart offered patients several features to manage care, such as access to after-visit and discharge summaries, outpatient test results, and the ability to update personal information, medications, allergies, and vaccinations. Patients can view their appointment schedule, electronically check in, cancel appointments, launch video visits, and request information for themselves or to share with other providers or people involved in their circle of care [5]. Though some health systems offer messaging between patients and providers through MyChart, THP had not activated this feature at the time of the study. At THP, MyChart functionalities were introduced in a staged manner, with new features launching every quarter. Before MyChart was implemented, THP did not have a patient portal, and patients were required to request the release of their health information from the Health Records department.

### Study Design

Six months after MyChart was first offered to patients, we conducted an explanatory sequential mixed methods evaluation [18] to understand health care providers’ perspectives about the initial impacts of MyChart on patient care and their workload and gather suggestions for improvement. We applied the RE-AIM (Reach, Effectiveness, Adoption, Implementation, Maintenance) framework and its extension to dimensions of sustainability and equity to guide our evaluation, to ensure a comprehensive assessment of MyChart’s impact and its potential for long-term sustainability and equitable outcomes [19,20]. We used a framework to guide the development of our questionnaire and interviews, focusing on key components of MyChart adoption and use. These components included effectiveness, such as provider-perceived impacts, negative outcomes, and patient engagement; adoption, such as provider comfort with using and promoting MyChart to patients; implementation, such as time spent by providers teaching patients about MyChart; and maintenance, such as provider capacity and resources for ongoing patient support with MyChart.

In phase 1, we conducted a cross-sectional web-based questionnaire via Qualtrics to explore health care providers’ experiences and satisfaction with using MyChart in clinical practice, perceived impacts on their workload and patient care, and suggestions for the future (refer to Multimedia Appendix 1). The questionnaire was pilot-tested by 7 health care providers and revised based on their feedback. In phase 2, we conducted individual semistructured qualitative interviews with health care providers to provide additional richness and depth to questionnaire data (refer to Multimedia Appendix 2).

### Participants

We recruited health care providers (physicians, nurses, and allied health) across the entire THP organization to complete a 5-minute web-based questionnaire. Health care providers were invited to participate through various institutional networks including an organization-wide email, weekly email newsletters, emails from clinical managers and directors in their departments, and daily email announcements.

For the semistructured interviews, health care providers who participated in the questionnaire were asked to indicate their interest in participating in a 30-minute follow-up interview at the end of the questionnaire. We used a combination of purposeful and maximum variation sampling to select health care providers with varying experiences, and perspectives about MyChart implementation in clinical practice [21,22]. This approach allowed us to recruit health care providers from various clinical programs with different clinical roles spanning a range of years of experience.

### Data Collection

The questionnaire consisted of closed and open-ended questions and was open throughout March 2024. Health care providers provided implied consent to participate after reading an informational sheet at the beginning of the questionnaire. Questionnaire items were mostly optional to gather as much data as possible, even if responses were partially complete.

Questionnaire participants who were interested in a follow-up interview completed one semistructured interview with a female mixed-methods researcher (ST). These were conducted over Zoom between March and May 2024. Participants provided verbal consent at the start of the interviews, during which they were asked to describe their experiences with using MyChart in clinical practice, identify challenges, and make suggestions for improvement. Interviews were audio-recorded, deidentified, and transcribed verbatim; transcripts were not reviewed by participants. Data collection occurred until the research team determined information power was reached, based on the study’s objective, the sample specificity, dialogue quality, and analysis strategy [23].

### Data Analysis

We used descriptive statistics to analyze data from closed-ended questions in the questionnaire. Quantitative data were analyzed using Microsoft Excel and Qualtrics and presented as counts and percentages.

We applied thematic analysis to analyze qualitative data from the open-ended questionnaire items and interviews [24]. First, we familiarized ourselves with the data through repeated readings of the transcripts. After, repeating readings, 2 researchers separately generated initial codes based on key patterns and concepts. ST and SV met to develop a draft codebook and applied it to a sample of 5 transcripts and open-ended responses. After initial coding, the codebook was refined and applied to the remaining transcripts. NVivo 12 (Lumivero) was used for data analysis and management.

### Ethical Considerations

This project was deemed quality improvement by the Trillium Health Partners Research Ethics Board and exempt from ethics board approval. Participants received an informational letter before completing the questionnaire and interview, outlining the study’s purpose, potential benefits and risks of participation, and procedures for data deidentification and use. Informed consent was obtained from all participants before the questionnaire and interview. Interview participants received a CAD $50 (US $35) honorarium in the form of a gift card for their time.

## Results

### Participant Characteristics

**Figure 1:**
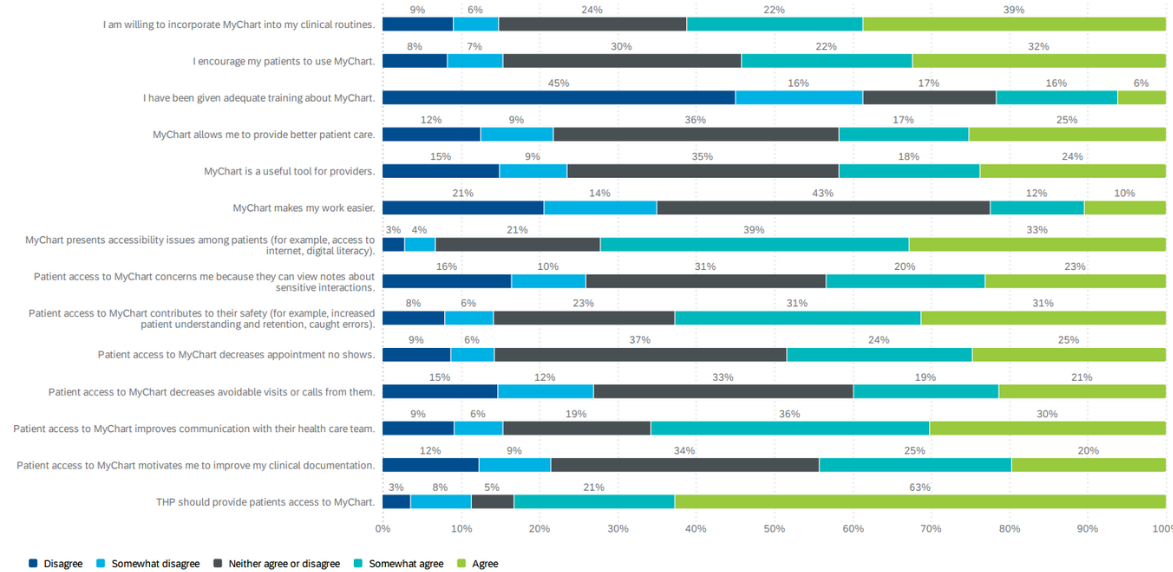
Questionnaire participants’ perceptions of the patient portal MyChart.

In total, 428 individuals completed the questionnaire. After excluding, 167 responses that were incomplete (more than 50% of the questionnaire was blank) or from staff who were not health care providers but mistakenly believed they were eligible to participate, the final sample consisted of 261 individuals. Among the questionnaire participants, 44% (106/242) were nurses, 29% (70/242) were physicians, and 27% (65/242) were allied health professionals. In total, 15 health care providers participated in follow-up interviews, including 4 physicians, 6 nurses, and 5 allied health professionals. Participant characteristics are described in Table 1, and a summary of their perceptions of and experiences with MyChart are shown in Figure 1. In both the open-ended items on the questionnaire and in interviews, participants shared their experiences with and perspectives about the initial impacts of MyChart, focusing on patient care, workflow integration, and usability. Results are summarized below across common themes from the quantitative and qualitative data

**Table 1:** Participant demographics, experience, and clinical background.

Characteristics^a^			Questionnaire, n (%)		Interviews, n (%)
**Age**					
	<25 years	4 (2)		0 (0)	
	25-34 years	37(15)		2 (15)	
	35-44 years	82 (33)		6 (46)	
	45-54 years	75 (30)		5 (38)	
	55 years or older	50 (20)		0 (0)	
**Roles**					
	Allied Health^b^	65 (27)		5 (33)	
	Nurse (registered nurse, nurse practitioner, registered practical nurse)	106 (44)		6 (40)	
	Physician	70 (29)		4 (27)	
	Physician’s assistant	2 (1)		0 (0)	
**Clinical area**					
	Cardiac health	22 (9)		0 (0)	
	Diagnostic imaging	4 (2)		0 (0)	
	Emergency department and urgent care	18 (7)		2 (13)	
	ICU^c^ and critical care	12 (5)		0 (0)	
	Inpatient medicine	20 (8)		1 (6.7)	
	Laboratory medicine and genetics	11 (4)		1 (6.7)	
	Mental health	20 (8)		1 (6.7)	
	Neuro or musculoskeletal	14 (6)		2 (13)	
	Oncology	30 (12)		3 (20)	
	Other (eg, clinical resource team)	11 (4)		0 (0)	
	Outpatient medicine and renal	23 (9)		2 (13)	
	Pharmacy	4 (2)		0 (0)	
	Primary, rehab, CCC^d^, palliative, senior	19 (8)		1 (6.7)	
	Surgery and Perioperative care	19 (8)		1 (6.7)	
	Women’s and children’s	19 (8)		1 (6.7)	
**Setting**					
	Ambulatory care	81 (34)		9 (60)	
	Inpatient care	88 (37)		2 (13)	
	Both ambulatory and inpatient care	70 (29)		4 (27)	

^a^Some participants did not report each characteristic.

^b^Allied Health included an occupational therapist, physiotherapist, pharmacist, dietitian, speech-language pathologist, social worker, radiation therapist, midwife, genetic counselor, dialysis assistant, kinesiologist, and child and youth worker.

^c^ICU: intensive care unit.

^d^CCC: complex continuing care.

### Patient Access to MyChart

Health care providers agreed or somewhat agreed that patients should have access to MyChart (215/258, 84%), as they perceived it to improve communication between patients and health care teams (168/255, 66%), and contribute to patient safety by increasing patient understanding of their own health and identifying errors (160/255, 62%). Among interview participants, those who strongly supported patient access to MyChart were often users of the tool themselves.

Obviously, you know, empowering patients with accessibility to their own information is really helpful and important. And, like, I have family members with chronic illness and it’s very helpful for me to be able to just look at their results...

Physician #2

Participants indicated that certain patient populations may have difficulty with accessing and using MyChart, such as those who are uncomfortable using technology (166/182, 91%), have limited access to devices (129/182, 71%) and internet (127/182, 70%), and experience language barriers (132/182, 73%). Additional populations with potential barriers to using MyChart included geriatric patients, children aged 12 years or younger, and individuals with cognitive impairments.

### Patient Access to Information Within MyChart

In this health system setting, MyChart features were made available to patients in a staged approach, where after-visit and discharge summaries, THP outpatient laboratory as well as diagnostic imaging reports, and appointment details were introduced as part of the initial implementation. E–check-in functionality and patient-entered questionnaires were phased sequentially over time. Health care providers had differing views on the staged approach of the release of information; while they tended to agree that it may have lessened upfront impacts on workflow and workload, there were varied perspectives about releasing additional information to patients in the future. Some advocated for releasing more test results and clinical notes, which was seen as enabling patients to share results with family members, understand their health, and seek second opinions.

More information should be available to the patient via MyChart - both inpatient and outpatient. it is important for patients to have access to their labs, clinical documentation, medications orders, as it gives them oversight into their care, opportunity to ask questions and catch errors or discrepancies. It gives them control over their health rather than providers dictating what should be done.

Pharmacist #1

Others voiced concerns that patients accessing results, images, and notes before health care encounters could heighten anxiety and create extra administrative work due to calls about “concerning results” and prompting expedited appointments. There were also concerns about patients viewing sensitive results (eg, diagnostic images) before discussing them with their healthcare provider or misinterpreting them.

But in terms of actually disclosing medical reports that are written in medical language with the intention of being a communication between medical providers, I think it results in unnecessary anxiety for patients frequently. I think there’s certain types of patients that will review the details of their clinical imaging reports or pathology reports, clinical notes, and they will, you know, be highlighting things and freaking out about things. Sometimes totally inappropriately just due to their not being a typical intended recipient of that type of documentation. Sometimes appropriately if there could be bad news but instead of in the supportive, hopefully supportive environment of a doctor patient interaction, by themselves, alone in their house with maybe many days before they are actually going to be reviewing that with their provider.

Physician 4

To address concerns about patient anxiety, multiple participants proposed releasing test results and notes only after health care encounters, which could allow patients to receive and interpret information in a supportive environment.

### Impacts on Patient Care

When asked if MyChart allowed health care providers to provide better care, 42% (108/255) agreed or somewhat agreed, while 36% (94/258) were neutral. One nurse described that MyChart provided patients with comfort, allowing them to get secondary opinions or explanations if needed.

It gives them comfort. That’s just sense I got. So, for some of the patients they just – they would ask, like, can I get this information, can I get this information. By having MyChart access they know that they could access at any time if they want to – like, if they want to talk to the family member or a family doctor or any other – anybody within, like, within their comfort level. Like, they want to share their information. They know that they could access it and then actually share medical charts. Instead of saying, oh, based on my memory this is what I was what I heard and what I told. Like, that’s not the case now.

Nurse #3

While health care providers identified some benefits to patients having MyChart, there were also concerns. In total, 43% (107/251) of participants agreed or somewhat agreed that patient access to MyChart concerned them as patients could view notes about sensitive interactions, while 31% (77/251) felt neutral about this. Despite patient health information being available for patients to access via a request to the Health Records department, the increased access to notes caused some to express concern that allowing patients to view notes that they may not agree with or that describe challenging interactions could have a negative impact on patient-provider relationships.

And so I’ve had them say, like, you know, can you correct this from this note [discharge summary], or I read this and I disagree with this. Or I’ve even had patients sort of get a little bit kind of offended, I guess. Because, like, in the mental status exam, for example, if you can say, like, poor insight into illness or having delusions of grandiosity so they could read that and then they come back and they’re a little bit sort of dysregulated because of that.

Nurse Practitioner #1

Although clinical notes were limited to just discharge summaries, some perceived negative impacts or raised concerns that allowing proxies to view certain records could cause negative consequences for patients.

We have something called an elder abuse screen which is, like, a piece of paper we fill out to follow a flowchart to determine what results should be taken in elder abuse. Like, should that be immediately available in my notes to a patient that an elder abuse screen was completed? Yes. But [...] The elder abuse screen is also used for people who are incapable. Right? Incapable in quotes. So, it would be their SDM [substitute decision maker] who would be accessing MyChart on their behalf. What if the SDM is the abuser and then they go and they access MyChart? And then they see that.

Social Worker #1

### Workflow Integration

In an effort to limit clinician workload in a time of health human resource crisis, THP had intentionally limited impact on clinicians during the launch of MyChart, particularly through its staged approach to releasing MyChart features over time. However, this has led participants to report inadequate training and information on MyChart and its available functionalities (116/258, 45%). This also may have contributed to our findings that 22% (58/258) of participants agreed or somewhat agreed that MyChart made their work easier, and 42% (108/258) agreed or somewhat agreed that MyChart is a useful tool for providers, and 68% (173/254) agreed or somewhat agreed that MyChart had no impact on their workload. A lack of knowledge about MyChart, along with new responsibilities to promote signing up for MyChart, caused some concern among health care providers about workflow, workload, and changing documentation practices. These views may have also been attributable to the limited MyChart features offered at THP; for example, there was no need to respond to patient messages and health care providers were not activating MyChart accounts. While some providers were not aware that MyChart was being implemented in a staged approach, others agreed that this method eased the need to immediately incorporate it into clinical routines, especially when they may not be familiar with all of its features and functionalities. Some interview participants reported having to provide detailed explanations for clinically irrelevant information; while they acknowledged the benefit of patients being informed about their health, in-depth explanations can lead to extended encounters especially if patients have limited health literacy.

Because with all of that extra information most patients are not – don’t have the health literacy to actually interpret the results or the test that they see. And so, you know, it can become challenging because they will ask questions about, you know, pertinent results that are contributing to the clinical picture, which is one thing, but then they also will often ask about minor changes or clinically relevant, you know, findings that we don’t necessarily consider... Sometimes I feel like I’m teaching an introductory, like, medical school class and that’s very burdensome when you have, like, a high volume of patients and you have – you know, sometimes these requests or these questions are coming from family who aren’t even there physically, like, there, calling in remotely saying that they found something. So that’s a challenge.

Physician #2

MyChart also changed workload by influencing clinical documentation practices. Among 253 participants, 45% (n=112) agreed or somewhat agreed that patients accessing MyChart motivated them to improve their clinical documentation to increase the accessibility of language for patients to read and understand. However, some expressed concerns about how adjusting their current documentation routine might impact workload in the future.

Though messaging between patients and providers had not been introduced as a MyChart feature in this health system, its hypothetical implementation was an area of tension. A few providers were in favor of it, but most were opposed, fearing it would increase uncompensated time to their workload and disrupt their workflow. Some raised concerns about patient expectations for immediate responses and suggested a need for clear guidelines for the type and timeliness of communication that would be required through messaging.

There’s pros and cons. So, the pro is, you know, as a patient you can – it’s better access to care. It’s better access to addressing any kind of issue or concern you may have, whether it be clarification about a prescription or an appointment or care that’s being provided. But I can see from a healthcare professional’s point of view where, you know, you might get inundated with, you know, these requests or emails or chats. And so, you know, what is the guideline in terms of, you know, what types of chats you should be receiving or what kind of information you can be sharing over the chat, whether it’s secure. And what’s sort of the guidelines in terms of when you’re expected to respond to something.

Nurse #1

## Discussion

### Principal Results

In this study, 261 health care providers completed a questionnaire, and 15 shared their perspectives about and experiences with the initial impact of implementing the MyChart patient portal at a large community hospital. Our findings revealed a mixed response. While most health care providers were generally supportive of providing patients access to their health information, limited health care provider engagement and clear communication about the role of MyChart in care may have negatively impacted how health care providers perceived the tool. Those who were MyChart users themselves had more favorable views of it and, logically, understood more about how it can support patient care. However, some providers had concerns about the potential negative consequences of patient access to MyChart, including patient anxiety or misunderstandings from viewing sensitive notes, increased provider workload due to additional explanations, and accessibility challenges for patients with limited technology skills, internet access, or language barriers. We identified an opportunity to strengthen health care providers’ understanding of how MyChart can be best integrated into their routines and the care they deliver; one unexpected and relatively simple way to achieve this could be to encourage them to access the portal themselves.

### Comparison With Previous Work

Participants raised concerns about patients or proxies viewing notes or results for sensitive interactions or diagnoses, which could be misinterpreted and then negatively impact patient-provider relationships. This aligns with other studies, which identified concerns among health care providers about patients accessing mental health notes or pathology test results [25-28], and becoming overwhelmed, hurt, or surprised by the content. Studies have also reported worries of unauthorized portal access, for instance, by a curious spouse reading a patient’s mental health notes detailing domestic abuse [25]. Concerns have also been raised about patients discovering new diagnoses from alerts on patient portals, leading to distress [28,29], which highlights the need for better communication about accessing sensitive information such as adding disclosures to alerts and notes. For instance, patients could be directed to indicate if they prefer to see results only after discussing with them with their health care team and opt out of viewing sensitive notes.

Interestingly, participants reported that MyChart improved patient-provider communication even in the absence of a secure messaging feature. This may possibly be due to both parties having clearer and more consistent access to patients’ health information, allowing for more meaningful, informed, and productive conversations. Though secure messaging through portals is often desired by patients [30], this was an area of concern for many providers in our study. They feared it could increase their already-high workload, disrupt their workflow, and was not something they would be explicitly compensated for. Secure messaging has been a conflicting debate in the literature, with some studies demonstrating that it does not tend to impact health care provider workload [13,31,32], as it can reduce in-person visits and follow-up calls and help patients self-manage; however, other studies have found that it can increase indirect patient care activities [26,33,34].

Introducing patient portals requires organizations to take a thoughtful approach that considers potential positive benefits, potential harms, manageable workloads for staff, and evolving regulatory landscapes (eg, the Cures Act in the United States) [35]. Our institution followed a staged approach to releasing MyChart features, which reflected organizational capacity and early engagement with health care providers suggesting limited readiness for comprehensive features to be introduced all at once, likely related to high care volumes, limited resources and support for equipping health care providers to rapidly adopt and proficiently use MyChart, and ongoing challenges with retention of health human resources as well as burnout post the COVID-19 pandemic [36]. Participants in our study agreed this approach likely mitigated some initial challenges of implementing technology into clinical environments; as many other health systems also face similar challenges, our peers may benefit from a similar approach to align with capacity limits and available resources.

Providers in this study reported a limited awareness of what patients could access in MyChart, and expectations for how it should be incorporated into care routines. However, those who were MyChart users themselves often had more favorable attitudes toward MyChart and would likely be able to provide better support to patients as they navigate the tool. Our health system also serves the most diverse patient community in Canada with 52% of residents identifying as immigrants and 62% as visible minorities [10], which participants agreed can introduce challenges around language and accessibility barriers. Comprehensive support for both patients and providers to learn about and access patient portals can help address apprehension about them, and challenges with digital navigation, and encourage patients to access their full range of features [26,37].

### Strengths and Limitations

The use of a mixed methods approach, combining both qualitative and quantitative data, allowed for a holistic and deep evaluation of perspectives and experiences with MyChart during the first 6 months it was available to patients. This approach, coupled with a diverse participant pool representing various clinical settings, roles, ages, and experience levels, ensured a rich and nuanced understanding of the impact of MyChart.

Our study is limited by a relatively low response rate to the questionnaire, despite a broad communication cascade and long duration, but this may have been attributable to limited engagement with health care providers upon MyChart’s launch. As a result, we were unable to interview participants from every clinical program in our health system to gain comprehensive perspectives about MyChart, as its use varies by clinical area; this may limit the transferability of our findings to areas where the portal is not widely used. However, our participants often represented clinical areas where MyChart is more frequently used by patients such as oncology and outpatient medicine. Our study was undertaken in the context of a staged release approach of portal features that followed the implementation of a hospital-wide digital health information system (Epic), so our findings may also not be transferable to health care systems using a different implementation strategy. Though community health care providers outside of tertiary care centers in our health system play important roles in delivering care, we did not include them in this study because MyChart was introduced in hospital settings only. Future studies will include their perspectives to understand how entire health systems interact with and support patient portals.

### Conclusion and Recommendations

A number of recommendations for peer health systems flow from our findings. Given the number of participants who reported inadequate training and knowledge of MyChart, it is important to engage providers early in the process of implementing a patient portal. This can help to understand their readiness and capacity for introducing a new tool into care and create clear alignment about its purpose and expectations for how health care providers will use it. Encouraging providers to sign up for accounts themselves (when applicable) and providing ample resources to learn about and use patient portals can help introduce the portal into care and reduce the time providers may need to spend assisting patients with technical difficulties during health care encounters. Participants in our study shared their concerns about secure messaging, especially around the increase in workload. To address these concerns and manage the tension that may exist between patient priorities and provider capacity, it is recommended to invite both groups to share dialogue around shared expectations for the content and timeliness of secure messaging communication. In addition, allowing physicians to bill for time spent messaging patients [38,39] could mitigate the risk of resistance to this feature or burnout. Finally, to reduce the number of repetitive patient questions about bloodwork or imaging and enhance patient education, health care systems should implement a system to track the queries health care providers receive. By identifying common themes and areas of confusion, resources can then be added to the portal to address these questions, making it easier for both patients and health care providers.

## Appendix

Multimedia Appendix 1 HealthCare Provider Questionnaire.

Multimedia Appendix 2 Interview Guide for Health Care Providers.

## Abbreviations

RE-AIM: Reach, Effectiveness, Adoption, Implementation, Maintenance
THP: Trillium Health Partners

## Data Availability

The data generated and analyzed during the current study are not publicly available. Participants did not consent to data sharing with individuals outside of the research team.
