# Supplementary material for: Examining Health Care Provider Experiences With Patient Portal Implementation: Mixed Methods Study
Source: J Med Internet Res. 2025 Jan 31;27:e65967. doi: 10.2196/65967 (PMC11829176; doi:10.2196/65967)
Supplement: Multimedia Appendix 1 [file jmir_v27i1e65967_app1.docx]

# Appendix – HealthCare Provider Questionnaire

Study Title: Evaluating MyChart activation, use, and experiences at Trillium Health Partners

By continuing with the questionnaire, I consent to participate. [click to launch questionnaire on next page]

1. Before today, did you know that THP patients may sign up for MyChart (a patient portal that allows them access to certain health information)? (yes/no)
2. Please select the most appropriate option: disagree/somewhat disagree/neither/somewhat agree/agree
   1. THP should provide patients access to MyChart.
   2. I have been given adequate training about MyChart.
   3. I am willing to incorporate MyChart into my clinical routines.
   4. MyChart allows me to provide better patient care.
   5. MyChart makes my work easier.
   6. MyChart is a useful tool for providers.
   7. I encourage my patients to use MyChart.
   8. MyChart presents accessibility issues among patients (for example, access to internet, digital literacy)
   9. Patient access to MyChart improves communication with their health care team.
   10. Patient access to MyChart contributes to their safety (for example, increased patient understanding and retention, caught errors).
   11. Patient access to MyChart decreases appointment no shows.
   12. Patient access to MyChart decreases avoidable visits or calls from them.
   13. Patient access to MyChart motivates me to improve my clinical documentation.
   14. Patient access to MyChart concerns me because they can view notes about sensitive interactions.

If selected 2d:

1. Please let us know the reason why MyChart presents accessibility issues among patients
   1. Language barrier
   2. Lack of comfort using technology
   3. Lack of access to the internet
   4. Lack of access to a device
   5. Other: free text
2. MyChart has
   1. Endpoint options: decreased my workload, somewhat decreased my workload, had no impact on my workload, somewhat increased my workload, increased my workload
3. I have suggestions about next steps or improvements to MyChart: free text

Demographics

This information is being collected so that we can better understand how to support all members of the Trillium Health Partners community. If you choose to answer the questions below, we will not be able to identify you or link your responses to this questionnaire back to your identity. All the information collected in this questionnaire is private and will be stored on THP’s secure server.

1. Age
   1. <25 years
   2. 25-34 years
   3. 35-44 years
   4. 45-54 years
   5. 55+ years
2. Role:
   1. Physician
   2. Physician’s assistant
   3. Nurse
   4. Nurse practitioner
   5. Registered practical nurse
   6. Occupational therapist
   7. Physiotherapist
   8. Pharmacist
   9. Dietitian
   10. Speech language pathologist
   11. Other (specify)
3. Please select the most appropriate clinical area where you work
   1. Cardiac Heath
   2. Diagnostic Imaging
   3. Emergency Department
   4. ICU and Critical Care
   5. Inpatient Medicine
   6. Laboratory Medicine & Genetics
   7. Mental Health
   8. Neuro/Musculoskeletal
   9. Oncology
   10. Outpatient Medicine & Renal
   11. Pharmacy
   12. Primary, Rehab, CCC, Seniors
   13. Surgery and peri-op
   14. Women’s and Children’s
   15. Other
4. Please select the most appropriate setting for your work
   1. Ambulatory care
   2. In-patient care
   3. Both ambulatory and In-patient care
5. We would like to learn more from health care providers about their experiences with MyChart in a short (up to 30 minute) interview. If you would like to learn more about participating in an interview, please click the link below, provide your email address, and we will follow up with you. Your email address will not be linked to your survey responses. Participants will be offered a $50 gift card for their time.

Interview for MyChart HCP - Survey

We would be delighted if you could kindly share your name, email and phone number with us. One of our team members may reach out to you to arrange a date and time that aligns with your schedule. Your cooperation is greatly appreciated, and we look forward to speaking with you.

Name:

Email:

Phone number:

# Appendix – Interview Guide for Health Care Providers

1. Can you tell me about a recent experience you had with MyChart? How did this change your experience as a health care provider from when THP wasn’t using MyChart?
2. Have you received the support you need to incorporate MyChart into your clinical routines? How comfortable are you with doing this?
3. How has MyChart impacted your day to day workflow? Can you provide specific examples?
4. How comfortable do you feel with helping patients activate or use MyChart? Is this a significant part of your clinical encounters?
5. Have you observed barriers to accessing MyChart among specific patient groups (e.g., older patients, patients who are not fluent in English)?
6. In your opinion, what are the key strengths of MyChart?
7. Since MyChart was launched in September 2023, THP has introduced some of its features (for example, scheduling, results release, patient-entered questionnaires, and billing) in a staged approach over time. Can you tell me about your experience with this approach?
8. Are there any areas where you believe MyChart could be further improved to better meet the needs of both [healthcare providers/staff] and patients? What specific enhancements or modifications would you suggest?
9. Is there anything else you’d like to tell me that we didn’t cover today?

Thank you so much for your participation. We’re very grateful to you for sharing your experiences.
