# Supplementary material for: Examining Health Care Provider Experiences With Patient Portal Implementation: Mixed Methods Study
Source: J Med Internet Res. 2025 Jan 31;27:e65967. doi: 10.2196/65967 (PMC11829176; doi:10.2196/65967)
Supplement: Multimedia Appendix 2 [file jmir_v27i1e65967_app2.docx]

# Interview Guide for Health Care Providers

1. Can you tell me about a recent experience you had with MyChart? How did this change your experience as a health care provider from when THP wasn’t using MyChart?
2. Have you received the support you need to incorporate MyChart into your clinical routines? How comfortable are you with doing this?
3. How has MyChart impacted your day to day workflow? Can you provide specific examples?
4. How comfortable do you feel with helping patients activate or use MyChart? Is this a significant part of your clinical encounters?
5. Have you observed barriers to accessing MyChart among specific patient groups (e.g., older patients, patients who are not fluent in English)?
6. In your opinion, what are the key strengths of MyChart?
7. Since MyChart was launched in September 2023, THP has introduced some of its features (for example, scheduling, results release, patient-entered questionnaires, and billing) in a staged approach over time. Can you tell me about your experience with this approach?
8. Are there any areas where you believe MyChart could be further improved to better meet the needs of both [healthcare providers/staff] and patients? What specific enhancements or modifications would you suggest?
9. Is there anything else you’d like to tell me that we didn’t cover today?

Thank you so much for your participation. We’re very grateful to you for sharing your experiences.
